# Performance of HIV pre-exposure prophylaxis indirect adherence measures among men who have sex with men and transgender women: Results from the PrEP Brasil Study

**DOI:** 10.1371/journal.pone.0221281

**Published:** 2019-08-20

**Authors:** Luana M. S. Marins, Thiago S. Torres, Iuri da C. Leite, Ronaldo I. Moreira, Paula M. Luz, Brenda Hoagland, Esper G. Kallas, José Valdez Madruga, Albert Y. Liu, Peter L. Anderson, Beatriz Grinsztejn, Valdilea G. Veloso

**Affiliations:** 1 Fundação Oswaldo Cruz, Instituto Nacional de Infectologia Evandro Chagas (INI/Fiocruz), Rio de Janeiro, Brazil; 2 Fundação Oswaldo Cruz, Escola Nacional de Saúde Pública (ENSP/Fiocruz), Rio de Janeiro, Brazil; 3 Universidade de São Paulo, Escola de Medicina, São Paulo, Brazil; 4 Centro de Referência e Treinamento em DST/AIDS, São Paulo, Brazil; 5 San Francisco Department of Public Health, Bridge HIV, San Francisco, California, United States of America; 6 Skaggs School of Pharmacy and Pharmaceutical Sciences, University of Colorado, Aurora, Colorado, United States of America; Washington University in Saint Louis, UNITED STATES

## Abstract

**Introduction:**

Efficacy of daily emtricitabine/tenofovir disoproxil fumarate (FTC/TDF) for PrEP is strongly dependent on the adherence. We examined the concordance between indirect adherence measures and protective drug levels among participants retained through 48 weeks in the PrEP Brasil Study.

**Methods:**

PrEP Brasil was a prospective, multicenter, open-label demonstration project evaluating PrEP provision for men who have sex with men (MSM) and transgender women (TGW) at higher risk for HIV infection within the setting of Brazilian Public Health System. Three indirect adherence measures were obtained at week 48: medication possession ratio (MPR), pill count and self-report (30-days recall). Tenofovir diphosphate (TFV-DP) concentration in Dried Blood Spot (DBS) was measured at week 48. Areas under (AUC) the receiver operating characteristics (ROC) curve were used to evaluate the concordance between achieving protective drug levels (TFV-DP≥700fmol/punch) and the indirect adherence measures. Youden’s index and distance to corner were used to determine the optimal cutoff points for each indirect adherence measure. We calculated sensitivity, specificity, negative (NPV) and positive (PPV) predictive values for the found cutoff points. Finally, Delong test was used to compare AUCs.

**Results and discussion:**

From April, 2014 to July, 2016, 450 participants initiated PrEP, 375(83.3%) were retained through 48 weeks. Of these, 74% (277/375) had TFV-DP ≥700fmol/punch. All adherence measures discriminated between participants with and without protective drug levels (AUC>0.5). High indirect adherence measure was predictive of protective drug levels (PPV>0.8) while low indirect adherence measure was not predictive of lack of protective drug levels (NPV<0.5). No significant differences were found between the adherence methods (p = 0.44).

**Conclusions:**

Low-burden measurements such as MPR and self-report can be used to predict PrEP adherence in a public health context in Brazil for MSM and TGW retained through 48 weeks.

Clinical Trial Number: NCT01989611.

## Introduction

Pre-exposure prophylaxis (PrEP) refers to the use of antiretroviral medications (ARV) by HIV-negative individuals aiming to reduce their risk of HIV infection. It is one of the most promising strategies in the field of biomedical HIV prevention [1]. The efficacy and safety of oral tenofovir disoproxil fumarate plus emtricitabine (FTC/TDF) as PrEP has been demonstrated in several trials [2–7]].

Cumulative evidence provided by iPrEX study[2], Partners PrEP [8] and TDF2 trial [6] led the United States Food and Drug Administration (FDA) to approve daily FTC/TDF for PrEP in July 2012 [9]. Now PrEP is available in numerous countries, including Brazil, where since December 2017, it has been offered as a public health policy to populations at high risk of acquiring HIV, as men who have sex with men (MSM) and transgender women (TGW) [10].

Brazil has been a pioneer in Latin America HIV/AIDS policies for both treatment and prevention [11], with universal free access to ARV and human rights-based prevention programs centered on behavioral and structural interventions [12]. Following the accumulated evidence on PrEP efficacy, since 2013 the Brazilian Ministry of Health (MoH) has supported projects on acceptability and feasibility of PrEP use in the country so as to generate evidence for the construction of the PrEP national policy [13]: PrEP Brasil [14]; Combina Project; PrEParadas Study; Horizon Project; Transgender Women Project Federal University of Bahia and ImPrEP [15]. PrEP demand was estimated to be 66,000–98,000 of Brazilian MSM aged 15–64 years [16] and from January to September 2018, 5,712 individuals initiated PrEP provided by the MoH [17].

Adherence is a critical component to PrEP effectiveness and high levels of PrEP use are essential to maximize the public health impact of this prevention strategy [18,19]. During PrEP Brasil study, the main barriers to PrEP adherence were forgetting doses, change in daily routine, pills shortage and not having pills available at the time of dose. The three main facilitators were associating PrEP with some daily activity, being engaged with PrEP and keeping the tablets in some visible place [20]. More recently, an online survey conducted among 11,367 Brazilian MSM found the main barriers to PrEP use to be related to information about PrEP such as fear of not being 100% protected against HIV, of side effects, and that ART would not work if infected. PrEP with no cost, access to free HIV test and access to personal PrEP counseling were the main facilitators of PrEP use [21].

A pharmacokinetic modeling study demonstrated the relationship between PrEP use and protective efficacy, showing that adherence is essential for maximizing the effectiveness of PrEP [3,22,23]]. However, accurate assessment of adherence is challenging. Existing methods of assessing adherence are limited for various reasons that have been well described in the literature [23–27]], and there is no gold standard for adherence measurement [24,28]. Drug concentration levels could be considered the most accurate measure to quantify adherence, but the high costs of available methodologies make it unfeasible for implementation in routine clinical practice, especially in a public health context [28].

In this study, we examined the concordance between three indirect adherence measures (medication possession ratio, pill count and self-report) and highly protective drug levels measured from dried blood spot (DBS) among participants retained through 48 weeks in the PrEP Brasil Study.

## Methods

As described in detail previously [14,29], PrEP Brasil was a prospective, multicenter, open-label demonstration project evaluating PrEP provision for MSM and TGW at higher risk for HIV infection in the context of the Brazilian Public Health System. Study design and 48-weeks’ results on retention, engagement, ethics and adherence measured by drug levels were published elsewhere [14]. All procedures were conducted according to the principles expressed in the Declaration of Helsinki. The present analysis was approved by INI-Fiocruz Review Board (#08405912.9.1001.5262).

Participants received a bottle containing 30 pills of FTC/TDF at enrollment and, subsequently, were provided with a sufficient supply of pills for daily treatment until the next study visit (weeks 4, 12, 24, 36 and 48). Study pharmacists instructed the participants to take one tablet daily and advised them to return FTC/TDF pills in the original bottle to the pharmacy, whether used or not. At each study visit, the pharmacists provided adherence support and counseling. At week 48, we measured adherence using three different methods: (a) medication possession ratio (MPR); (b) pill count; and (c) self-report.

MPR reflects the days the participant is “covered” by the study medication. It is calculated by the ratio of the number of pills dispensed at the prior visit and the number of days between that visit and the week 48 visit. MPR values equal or greater than 1.00 indicate 100% coverage, and values below 1.00 reflect that the participant was not covered by the study medication during all the days between week 48 and the prior visit [30].

Pill count was calculated by the number of pills dispensed at the prior visit minus the number of pills returned at week 48, divided by the number of days between the two visits. Values are given in percentage. We could not calculate pill count for the participants who did not return any bottle of study medication for counting at week 48. These cases were considered missing data and not included in the accuracy analysis (n = 42).

Self-report was assessed using one question from a structured questionnaire (“**On average for how many days did you forget to take FTC/TDF in the previous 30 days?**”) administered by study pharmacists. Values are given in percentage. Participants attending the week 48 visit who refused to answer the questionnaire (n = 28) were considered missing and not included in the accuracy analysis.

DBS specimens were collected for tenofovir diphosphate (TFV-DP) assessments for all participants who attended the week 48 visit. TFV-DP levels were measured using liquid chromatography-mass spectrometry tandem mass spectrometry (LC-MS/MS) at the University of Colorado Antiviral Pharmacology Laboratory (Aurora, CO, USA) with standard procedures [3,31,32]].

Results from iPrEX Open-label study demonstrated the following correspondence of doses taken per week, TFV-DP concentration and levels of protection: a TFV-DP concentration ≥700 fmol/punch, corresponding to ≥4 doses/week, was associated with a 100% (95% CI 86% to 100%) reduction in HIV transmission risk; and a TFV-DP concentration of 350–699 fmol/punch, corresponding to 2–3 doses/week, was associated with a 86% (21% to 99%) reduction in HIV transmission risk [3]. For the purposes of this analysis, TFV-DP levels were dichotomized as highly protective drug levels (≥ 700 fmol/punch) vs. poorly protective drug levels (<700 fmol/punch) [33].

The area under the curve (AUC) was estimated using a receiver operating characteristics (ROC) curve analysis to evaluate the accuracy of each indirect adherence measure in discriminating between those with or without highly protective drug levels. From the ROC curve, the optimal cutoff points for discriminating between those with or without protective drug levels were found based on the Youden index and the distance to corner [34–36]. Sensitivity, specificity, negative predictive value (NPV) and positive predictive value (PPV) for the cutoff points were calculated. Statistical comparisons of the AUC for each adherence measure were performed with the DeLong test, a nonparametric method commonly used to identify differences among AUC [37]. This method does not assume the strong normality assumptions that the alternative Binormal method makes. Analyses were conducted using SAS version 9.4 (SAS Institute, North Carolina, USA).

## Results

From April 2014 to July 2016, 450 participants initiated PrEP, of whom 375 (83%) were retained for 48 weeks. Table 1 depicts baseline characteristics for the 375 participants included compared with the 75 participants not included in this analysis. The full baseline characteristic for these participants are presented in previous work by the authors [14]]. At week 48, 277 (74%) of the 375 participants had highly protective drug levels consistent with at least four doses per week [14]]. Median adherence rates were 1.10 (IQR 0.98–1.31) for MPR, 97.14% (IQR 88.89–100.00) for pill count and 100.00% (IQR 93.33–100.00) for self-report. Among 42 participants who did not return any bottle of FTC/TDF for pill count, 35.7% and 64.3% had high and low protective drug levels, respectively. As for the 28 participants who did not complete the self-report questionnaire, 28.6% and 71.4% had high and low protective drug levels, respectively.

**Table 1:** Baseline characteristics of participants included in this analysis vs. not included.

	Total	Included n (%)	Not included n (%)	p-value
**Overall**	450	375 (83)	75 (17)	
**Site Location**				
RJ	180	150 (83)	30 (17)	1.00
SP	270	225 (83)	45 (17)	
**Age**				
18–24 years	113	92 (81)	21 (19)	0.52
25–34 years	214	179 (84)	35 (16)	0.83
≥35 years	123	104 (85)	19 (15)	
**Schooling**				
< 12 years	115	93(81)	22(19)	0.41
≥ 12 years	335	282(84)	53(16)	
**Color/Race**				
White	243	205(84)	38(16)	
Black	57	47(82)	10 (18)	0.72
Mixed	145	118 (81)	27 (19)	0.45
**Gender**				
Male	425	354 (83%)	71 (17%)	0.93
Transwomen	25	21 (84%)	4 (16%)	
**Steady partner**				
Yes	233	194 (83%)	39 (17%)	0.87
No	204	171 (84%)	33 (16%)	

**Fig 1:**
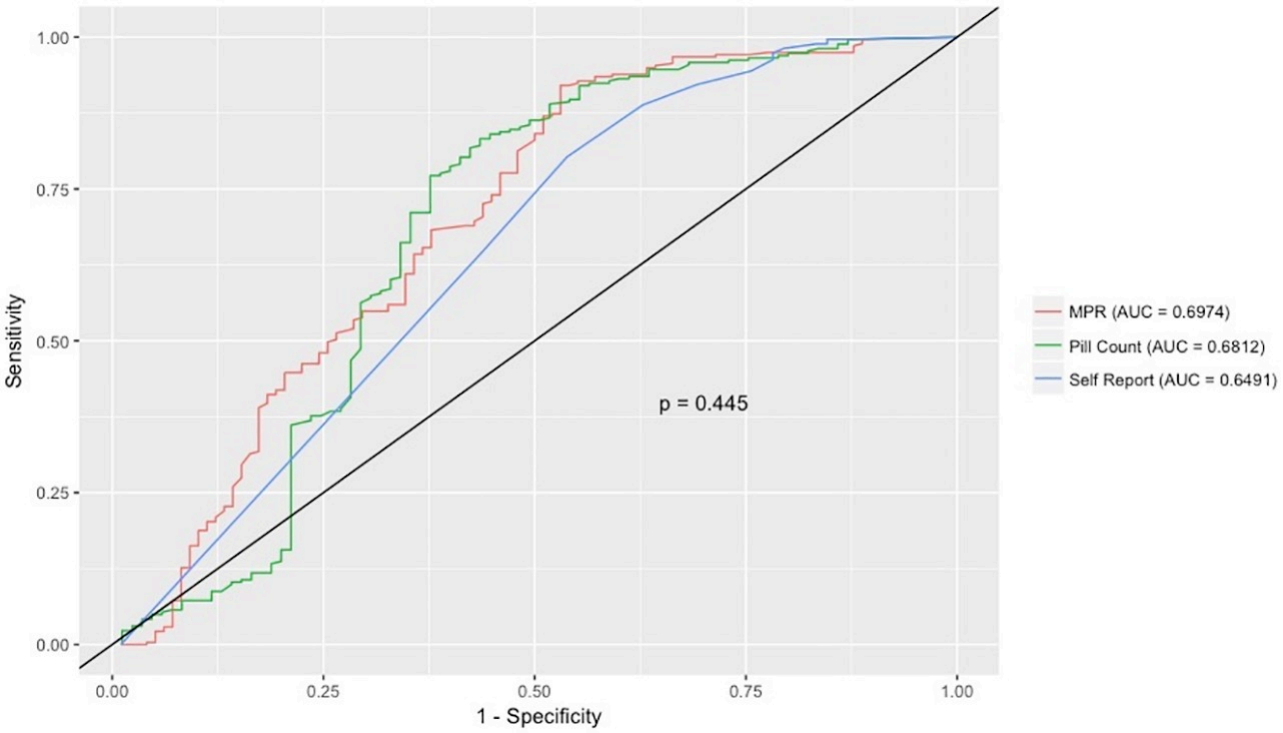
ROC curves for protective drug level vs indirect adherence measures. ROC curves of indirect adherence measures to predict protective drug levels. (*) P-value refers to the difference between the AUC of the three indirect adherence measures (De Long’s test).

According to the ROC curves, the three indirect adherence measures can predict highly protective drug levels at week 48 (Fig 1). The area under the ROC curve was 0.70 for MPR, 0.68 for pill count and 0.65 for self-report.

The cutoff value associated with highly protective levels of TFV-DP according to the highest Youden index was 1.07 for MPR, 90.09% for pill count and 99.90% for self-report (Table 2). The proportion of participants with adherence values equal to or higher than the cutoff point was as follows: 62.9% for MPR (236/375); 72.4% for pill count (241/333); and 59.6% for self-report (207/347).

**Table 2:** Association between indirect adherence measures and protective drug levels.

Adherence measures	AUC (95% CI)	P-value	Cutoff point	Sensitivity	Specificity	PPV	NPV
MPR	0.70(0.63–0.76)	<0.001	1.07	0.68	0.62	0.84	0.41
Pill count	0.68(0.60–0.76)	<0.001	90.09	0.77	0.62	0.86	0.47
Self-report	0.65(0.58–0.72)	<0.001	99.90	0.64	0.56	0.84	0.31

MPR = medication possession ratio; PPV = positive predictive value; NPV = negative predictive value.

The concordance between the adherence methods, in terms of positive predictive values (PPV) and negative predictive values (NPV), is depicted in Table 2. For all indirect adherence measures, high indirect adherence measures were predictive of protective drug levels (PPV>0.8), while low recorded adherence was not predictive of a lack of protective drug levels (NPV<0.5).

Comparison using the DeLong test showed no significant differences between the three indirect adherence measures in the ability to discriminate participants with and without protective drug levels (p = 0.44).

## Discussion

The main purpose of this study was to assess the concordance between a pharmacologic measure of exposure and “traditional”, feasible and low-cost adherence measures (pill count, medication possession ratio and self-report). This is the first study to evaluate the correlation of indirect adherence measurements in the context of a public health program in a middle-income country. As previously mentioned, PrEP efficacy is strongly dependent on adherence, thus, the identification of whether an individual in a PrEP Program is adherent or not is of great importance in the clinical setting. Each adherence measure has its strengths and weaknesses [26,27].

We observed high levels of adherence among participants retained through 48 weeks in PrEP Brasil study using all indirect adherence measurements (MPR, pill count and self-report) as well as by TFV-DP levels [14]. High adherence levels based on drug levels diverge from previous placebo-controlled PrEP trials [2,28,38,39], but such levels are consistent with findings from recent open-label and demonstration projects [28,40–43]. This result was expected, as people feel more comfortable using PrEP once more safety and efficacy results become available, and when such results are obtained outside a placebo-controlled study [28,44]. We observed low levels of adherence based on drug levels for those who did not return their bottles for pill count and for those who chose not to answer the self-report measure. Although a great effort was made to perform these assessments in a neutral supportive environment without judgment or direct persuasion, we suppose, given their low levels of adherence, that these individuals wanted to avoid any discomfort or fear to report socially undesired behavior.

Our results showed that all indirect adherence measures could discriminate participants with and without protective drug levels with fair discriminatory ability (AUC>0.5) among participants retained through 48 weeks. Additionally, there were no differences between the three indirect adherence measures in the ability to discriminate between participants with and without protective drug levels. The efficacy of adherence measures in discriminating between achieving or not achieving protective drug levels vary among clinical trials, a finding that can be explained by the differences in the study location and demographic characteristics. The results from iPrEx placebo-controlled trial which included 510 participants in 6 countries (Brazil, Ecuador, Peru, South Africa, Thailand and US) showed that only MPR was able to discriminate, with relatively poor discriminatory ability [30]. The Partners PrEP ancillary adherence study among East African serodiscordant couples also found poor discriminatory ability for self-report (AUC 0.56 [95% CI, 0.47–0.61]) and pill count (AUC 0.58 [95% CI, 0.46–0.60]) [42]. Likewise, the ATN-123 found that self-report could not accurately capture true levels of medication adherence among young MSM in US [45]. Nonetheless, the TDF2 PrEP trial in Botswana found a modest correlation between TFV drug levels and self-reported adherence (phi-coefficient = 0.28), and a weaker relationship between drug levels and pill count adherence (phi-coefficient = 0.20) [46]. The iPrEx open-label extension also found concordance for self-report with PPV and NPV of 83% (95% CI, 81.3–84.3) and 82% (95% CI, 81.3–84.3), respectively [47]]. We also determined the best cutoff points to discriminate high levels of adherence. Considering these cutoff values, PPV values for the three indirect adherence measurements were higher than 0.80. This means that approximately 80% of individuals with adherence higher than the cutoff values also have high adherence based on TFV-DP levels; thus, high recorded adherence was predictive of highly protective levels. Although all indirect adherence measures presented high PPV, it is important to emphasize that the possibility of “false positive” responses must be considered within clinical practice, once individuals presenting high indirect adherence measure may not achieve highly effective level of protection and thus be at risk of HIV. This could prevent the access of these individuals to adherence support (adherence counseling, for example). The use of 2 or more adherence measures may be useful to identify more efficiently low adherence in “real world”, as discussed in the “WHO consultation on PrEP adherence” [48]. Abaasa et al.[49] demonstrated the incremental value of combining adherence measures (electronic monitoring, self-report, and drug concentrations in plasma and hair). However, the authors used objective adherence measures, so more studies are needed on how the use of two or more indirect adherence measure can give an accurate picture of PrEP adherence.

Conversely, NPV values were low (<0.5), indicating that low recorded adherence was not predictive of a lack of protective drug levels. This finding can be explained by the fact that a highly protective drug level (≥700fmol/punch) can be achieved with less frequent dosing (4 pills/week) than the current approved recommendation of daily PrEP. Even those with low measured adherence may still have protective drug levels. It means that for achieving highly effective levels of protection (TFV-DP≥700fmol/punch) the average adherence over the period must be approximately 57% (4 taken doses/7 days = 0.57). It is important to note that the best cutoff points estimated in our study (1.07; 90.09% and 99.90%, for MPR, pill count and self-report, respectively) are higher than 57%, thus it is plausible that an individual with adherence lower than a given cutoff point could still achieve levels of protection.

Self-report is the most common adherence measure in clinical practice [50]. The major strengths of this measure are ease of collection, low cost and the accuracy of reported non-adherence [26]. Conversely, it depends on the participants’ memory or comfort in providing truthful information [51], thus this measurement is subject to bias (recall and social desirability bias) which often lead to overestimation of adherence. A way to improve self-report adherence is providing an environment where the individual feels at ease and confident in reporting socially undesired behavior [26]. Pill count is a more objective adherence measure and more accurate when compared to self-report. This measure is also easy to collect and relatively inexpensive [26], though, as limitations, it is time consuming and subject to manipulation (for example, pills discarded, lost or shared with others) and might not reflect the pattern of PrEP use [27,52]. MPR is derived through dispensation records, it corresponds to the percentage of days during which individuals are in possession of their medications [53] reflecting the pill coverage during a given time. Similarly to pill count, MPR do not provide PrEP pattern use as it indicates pharmacy dispensation rather than drug ingestion. MPR above 1.0, for example, does not necessary mean over adherence, but indicates that an individual possesses enough medication to cover a given time of PrEP use. According to “WHO consultation on PrEP adherence”, pharmacy refill “may be interpreted as maximal predicted PrEP adherence” [48]. MPR may be a powerful tool for adherence assessment, especially if dispensation information is accurate and accessible. The Brazilian experience with SICLOM (the National Computerized System for the Control of Drug Logistics)—which contains specific information about antiretroviral dispensation, including PrEP—suggests that this type of database could provide a unique data source for adherence assessment in the country and perhaps elsewhere [54]].

The present study has some limitations. First, the different time periods associated with each indirect adherence measure assessment (30-day recall for self-report, 90-day recall for pill count and MPR) and TFV-DP assessment (a 17-day half-life corresponding to cumulative adherence over 1–3 months) may impact comparisons. It is a common limitation when comparing a pharmacokinetic measure with a non-pharmacokinetic measure [30,42,49]. TFV-DF levels in DBS has a long half-life of 17 days, which provides an average adherence of 1 to 3 months[31,48], as red blood cells live in blood circulation for 100 to 120 days [55]. However, neither TFV-DF levels nor indirect adherence measures (MPR, pill counts and self-report) can provide patterns of FTC/TDF scheduling use over the mentioned period. Despite the different periods of assessment, the rationale for this analysis was to seek evidence on whether the three indirect adherence measures could or not discriminate participants that achieved highly-effective level of protection by using at least an average of 4 doses/week of PrEP, independently of the FTC/TDF scheduling use. Furthermore, it is already well established that through the determination of drug levels of TFV-DF in DBS we are able to assess the adherence “categories” of a given individual (7 doses/week; ≥ 4–7 doses/week; 2–3 doses/ week and <2doses/week). These categories have been used in PrEP demonstration projects to estimate gradients of adherence [56]. Second, TFV-DP levels were dichotomized as highly protective drug levels or poorly protective drug levels, limiting the interpretation of correlation data from this analysis. Third, drug levels were assessed for participants attending the week 48 visit, thus we assessed the performance of indirect adherence measures only for these participants. Though this limitation could have led to selection bias, we argue that, if any, the impact was minimal. We have previously reported that there was no significant difference on baseline characteristics between participants retained and not retained at week 48. Importantly, there was no difference in the levels of TFV-DP measured at week 4[29]. Fourth, the concordance between the indirect adherence measures was only obtained for the best cutoff points according to the aim of this study. For our analysis, we chose to give equal weight to sensitivity and specificity. Youden’s index and distance to corner were then used, and each sensitivity and specificity combination in the output was considered. Lastly, the low levels of adherence based on drug levels for those considered missing for pill count and self-report may have led to an overestimation in the discriminatory ability of these indirect adherence measures.

## Conclusions

In conclusion, this study provided the opportunity to assess the concordance between drug exposure and other indirect adherence measures in a real-world public health PrEP program. Our results highlight the utility of low-burden measurements such as MPR and self-report in predicting adherence among our target population. These measurements can be useful in monitoring PrEP use among MSM and TGW retained in a PrEP program and in guiding the need for adherence interventions. Studies on incremental value of combining MPR and self-report in predicting adherence among our target population are needed.
